# Missing-linker metal-organic frameworks for oxygen evolution reaction

**DOI:** 10.1038/s41467-019-13051-2

**Published:** 2019-11-06

**Authors:** Ziqian Xue, Kang Liu, Qinglin Liu, Yinle Li, Manrong Li, Cheng-Yong Su, Naoki Ogiwara, Hirokazu Kobayashi, Hiroshi Kitagawa, Min Liu, Guangqin Li

**Affiliations:** 1 MOE Laboratory of Bioinorganic and Synthetic Chemistry, Lehn Institute of Functional Materials, School of Chemistry, Sun Yat-Sen University, 510275 Guangzhou, China; 2 State Key Laboratory of Powder Metallurgy, Institute of Super-microstructure and Ultrafast Process in Advanced Materials, School of Physics and Electronics, Central South University, 932 South Lushan Road, 410083 Changsha, Hunan China; 3 Division of Chemistry, Graduate School of Science, Kyoto University, Kitashirakawa-Oiwakecho, Sakyo-ku, Kyoto, 606-8502 Japan; 4 JST, PRESTO, 4-1-8 Honcho, Kawaguchi, Saitama 332-0012 Japan

**Keywords:** Catalyst synthesis, Electrocatalysis, Metal-organic frameworks

## Abstract

Metal-organic frameworks (MOFs) have been recognized as compelling platforms for the development of miscellaneous applications because of their structural diversity and functional tunability. Here, we propose that the electrocatalytic properties could be well modified by incorporating missing linkers into the MOF. Theoretical calculations suggest the electronic structure of MOFs can be tuned by introducing missing linkers, which improves oxygen evolution reaction (OER) performance of the MOF. Inspired by these aspects, we introduced various missing linkers into a layered-pillared MOF Co_2_(OH)_2_(C_8_H_4_O_4_) (termed as CoBDC) to prepare missing-linker MOFs. Transmission electron microscope and synchrotron X-ray measurements confirmed that the missing linkers in the MOF could be introduced and well controlled by our strategy. The self-supported MOF nanoarrays with missing linkers of carboxyferrocene exhibit excellent OER performance with ultralow overpotential of 241 mV at 100 mA cm^−2^. This work opens a new prospect to develop efficient MOF-based electrocatalysts by introducing missing linkers.

## Abstract (web summary)

While water splitting electrocatalysis provides a means to store electrical energy as fuel, the water oxidation catalysts typically show low performances. Here, authors employ metal-organic frameworks with missing-linkers as highly active oxygen evolution electrocatalysts.

## Introduction

Developing efficient electrochemical conversion processes is of great significance for storing and utilizing renewable energy^1^. Electrochemical oxygen evolution reaction (OER) plays an essential role in many energy conversion technologies involving metal–air batteries, water splitting, and CO_2_ reduction^2–5^. Unfortunately, the efficiency of OER was limited by its sluggish kinetics and high over-potential. So, efficient electrocatalysts are highly required to facilitate OER effectively^6^. To date, noble metal catalysts including RuO_2_ and IrO_2_, have been recognized as effective electrocatalysts for OER^7^. Nevertheless, the large-scale application of noble metal catalysts was hindered by its high-cost and scarcity. Therefore, exploring new high-efficiency non-noble metal electrocatalysts for OER is of ongoing interest^8^. Although tremendous efforts have been devoted to developing cost-effective OER catalysts, current electrocatalysts still failed to satisfy the industrial requires. So, the development of non-noble metal electrocatalysts for OER with high catalytic activity and stability is still a challenge^9^.

Metal-organic frameworks (MOFs), have served as the fascinating material platform with versatile applications including gas storage and separation^10–12^, drug delivery^13^, and catalysis^14–19^. Benefiting from their isolated active site, large surface area, and high porosity, MOFs have received broad research interest in the field of heterogeneous electrochemical catalysis^20–24^. Nevertheless, most of MOFs show intrinsic poor electric conductivity and electrocatalytic activity. Although there have been some methods, such as metal node engineering^25,26^, hydroxide ligands cooperate^27^, and lattice-strained MOF^28^ reported to regulate the electrocatalytic activity of MOFs, the directly use of MOFs as efficient OER catalysts is still in its infancy.

The electrocatalytic performance of solid materials is mainly reflected by the number of active sites, electronic conductivity, and the reaction energy barrier of the catalyst^29^. In addition to regulate the morphology and crystal structure of the catalyst, optimizing the electronic structure of catalytic metal is the most straightforward way to change intrinsic characteristics of catalysts such as electronic conductivity and the reaction energy barrier^30^. The electronic structure of MOFs is mainly affected by topological structure and coordination environment^31^. Owing to the high design flexibility of MOFs, the missing linkers can be controllably introduced into MOFs by partially substituting multicoordinating bridging linkers with nonbridging ligands to change their coordination environment without loss of crystallinity and porosity of materials^32–35^. So, incorporating missing linkers into MOFs provides a promising strategy to tailor electronic structure of MOFs^36,37^. This may open up new opportunities for regulating the electrocatalytic property of MOFs.

**Fig. 1:**
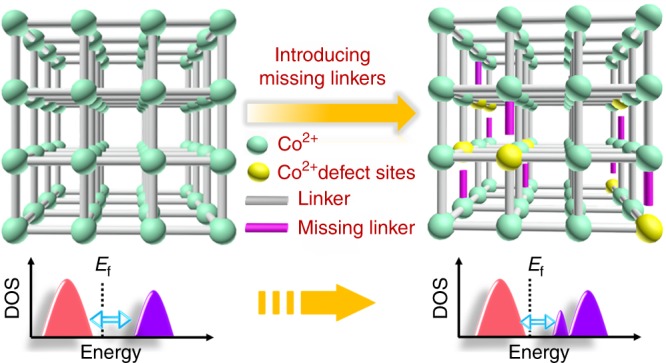
Modulating electronic structure of MOFs via introducing missing linkers for efficient OER

Therefore, we introduce missing linkers to regulate electronic structure of MOFs and report a universal strategy to enhance the OER activity of MOFs (Fig. 1). Theoretical results demonstrate that the electronic structure of CoBDC can be regulated effectively by incorporating missing linkers such as carboxyferrocene (Fc) into MOFs, changing the band gap and charge distribution thus optimizing adsorption strength for the reaction intermediates. In subsequent experiments, the MOFs with missing linkers exhibited an enhanced catalytic activity, validating our design.

## Results

### Density functional theory (DFT) calculations

**Fig. 2:**
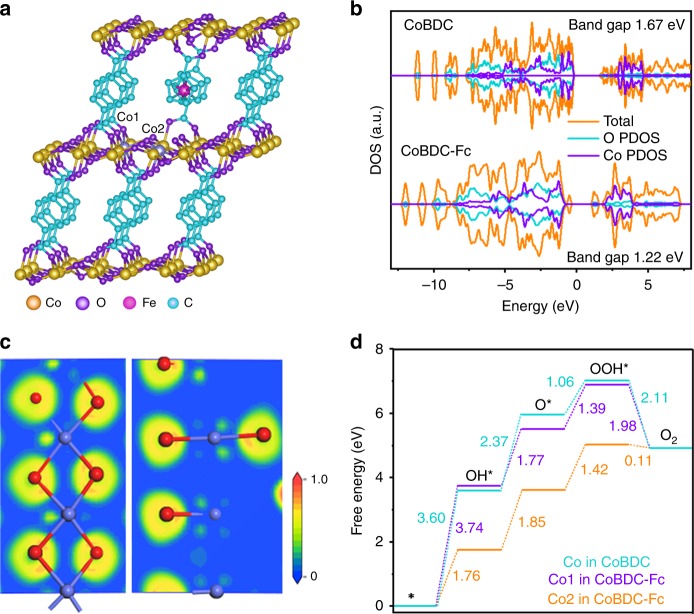
DFT calculations on improving OER performance of MOFs. **a** Crystal structure of CoBDC-Fc obtained from DFT simulation. **b** Calculated DOS of CoBDC and CoBDC-Fc. **c** Electron localization function of CoBDC-Fc. **d** Free energy diagram for OER on CoBDC and CoBDC-Fc

The layered-pillared MOF Co_2_(OH)_2_(C_8_H_4_O_4_) (named as CoBDC) constructed by the coordinated octahedrally divalent cobalt and terephthalic acid (H_2_BDC). The terephthalates are coordinated and pillared directly to the cobalt hydroxide layers and form a three-dimensional framework (Supplementary Fig. 1)^38^. owning to its unique structure and good stability^25^, CoBDC is used as an example to study the effects of missing linkers on its electronic structure. DFT reveals that the terephthalic acid in CoBDC can be repalced by missing linker of carboxyferrocene (Fc) and forms a new stable MOF named by CoBDC–Fc (Fig. 2a, Supplementary Fig. 2). After introducing missing linkers, CoBDC–Fc generates new electronic states near the Fermi level, suggesting a more conductive electronic structure (Fig. 2b). The partial density of states (PDOS) showed that the generation of new electronic states near the Fermi level can be ascribed to the change of electronic structure of Co and O. 2D electron localization function (ELF) analysis showed that a larger ELF value around the Co atom in CoBDC–Fc compared with that of CoBDC can be observed (Supplementary Fig. 3), indicating a higher electron localization on Co in CoBDC-Fc (Fig. 2c).

Free energy difference (Δ*G*) for each elementary step was calculated to estimate the OER activity on different sites. The optimized pathways of various sites were shown in Supplementary Fig. 4. Based on the free energy diagram (Fig. 2d) of CoBDC, the energy barrier (∆*G*1 = 3.74 eV) for the formation of OH∗ is the rate-determining step on the Co site of bulk phase (Co1 in CoBDC–Fc), which is assigned to the weak adsorption energy of OH* (Supplementary Table 1). The Co in bulk phase of CoBDC–Fc showed similar activity with Co in bulk phase of CoBDC. After introducing missing linkers into CoBDC, the defect site (Co2 in CoBDC–Fc) generated and enhanced the adsorption energy of the OER intermediates. As a result, the rate-determining step for CoBDC–Fc is the oxidation of OH* to O* with a smaller energy barrier of 1.85 eV. The decrease of energy barrier after incorporating missing linkers into MOF catalyst implies that the unique electronic structure in the defect site of the MOF plays a vital role in improving OER activity.

### Synthesis and characterization of missing-linker MOFs

**Fig. 3:**
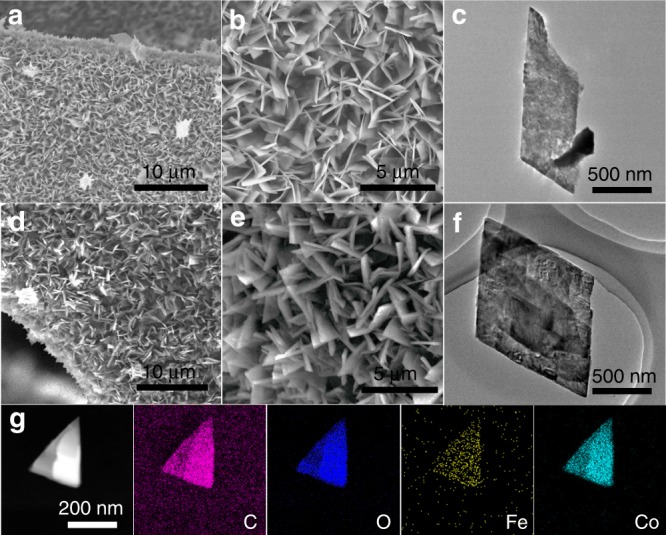
Physical characterization of CoBDC–Fc–NF. **a** and **b** SEM images of CoBDC–NF. **c** TEM image of CoBDC–NF. **d** and **e** SEM images of CoBDC–Fc–NF. **f** TEM of CoBDC–Fc–NF. **g** HAADF-STEM image and STEM-EDX mappings of CoBDC–Fc–NF

In light of DFT results, Fc was introduced into CoBDC to construct MOFs containing missing linkers (named as “CoBDC–Fc_*x*_” where *x* = the molar ratio of Fc:BDC) by modulation approach. In order to improve the electric conductivity and mechanical stability of MOF, self-supported CoBDC nanoarrays (CoBDC–NF) were prepared by reacting H_2_BDC with Co(NO_3_)_2_·6H_2_O in the presence of nickel foam (NF) substrate. The appropriate amount of Fc was introduced into CoBDC nanoarrays to construct defective MOF arrays (CoBDC–Fc–NF). The ratio of BDC:Fc was about 6:1 in CoBDC-Fc_0.17_ and CoBDC–Fc–NF determined by the measurement of inductively coupled plasma mass spectrometry (ICP–MS) (Supplementary Table 2). X-ray diffraction (XRD) patterns showed that the crystal structure of CoBDC is identical with the previously reported Co_2_(OH)_2_(C_8_H_4_O_4_) MOFs (Supplementary Fig. 5)^38^. The targeted incorporation of missing linker defects led to the formation of highly crystalline CoBDC-Fc_0.17_ which have almost the same diffraction patterns as CoBDC. The morphologies of as-prepared materials were investigated by scanning electron microscopy (SEM). Both CoBDC and CoBDC-Fc_0.17_ showed nanosheets morphologies (Supplementary Fig. 6). Employing NF as a substrate, CoBDC nanosheets can be uniformly grown on NF and form nanosheet array (Fig. 3a, b). After incorporating Fc as a modulator, CoBDC–Fc–NF shows similar morphology to CoBDC–NF (Fig. 3d, e). While CoBDC–Fc–NF was assembled by slightly thicker MOF nanosheets, compared to CoBDC–NF. Transmission electron microscopy (TEM) images further confirmed their nanosheet morphologies (Fig. 3c, f). Energy-dispersive X-ray spectroscopy (EDX) mappings demonstrate that the Fc can be uniformly incorporated into CoBDC nanosheets (Fig. 3g). The N_2_ sorption isotherms of as-prepared MOFs at 77 K showed that CoBDC–Fc_0.17_ had a smaller Brunauer–Emmett–Teller (BET) surface area (16.03 m^2^ g^−1^) than that of CoBDC (17.12 m^2^ g^−1^) (Supplementary Fig. 7).

**Fig. 4:**
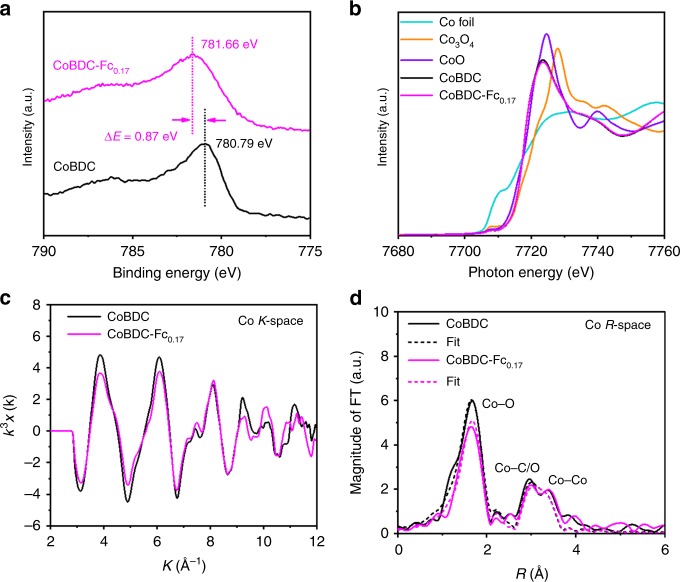
Electronic structure characterization of CoBDC–Fc_0.17_. **a** Co 2*p* 3/2 of CoBDC and CoBDC–Fc_0.17_. **b** Co K-edge XANES data of CoBDC, CoBDC–Fc_0.17_, and reference samples. **c** Co K-edge EXAFS of oscillations. **d** Fourier transformed EXAFS spectra of CoBDC and CoBDC–Fc_0.17_

In order to experimentally confirm the impact of introducing missing linkers on the electronic states of MOFs, X-ray photoelectron spectroscopy (XPS) was carried out to investigate the electronic structure of the active center. In the full range XPS spectra of CoBDC, the peaks of C1*s*, O1*s*, and Co 2*p* are detected. Introducing the Fc as modulator led to an obvious peak of Fe 2*p* in the XPS spectra of CoBDC–Fc_0.17_ (Supplementary Fig. 8). The Co 2*p*3/2 of CoBDC and CoBDC–Fc_0.17_ demonstrated that Co cation was bivalent Co^2+^ state (Fig. 4a). Compared with those of CoBDC, Co 2*p*3/2 and O1*s* in CoBDC–Fc_0.17_ have higher binding energy and broadened peaks, indicating the change of active center coordination environment caused by the introduction of missing linkers (Supplementary Fig. 9a)^39^. Furthermore, XPS valence band spectra were measured to investigate the electronic properties of the MOFs catalysts. As shown in Supplementary Fig. 9b, after introducing missing linkers, the valence band maximum energy of CoBDC–Fc_0.17_ blue shifts to the vacuum level at about 0.37 eV with respect to that of CoBDC (1.65 eV), suggesting that introducing missing linkers can effectively modulate the electronic structure of MOFs. Square resistance measurements proved that the conductivity of the MOF increased after introducing missing linkers (Supplementary Table 3).

To understand the local structures of Co^2+^ in CoBDC and CoBDC–Fc_0.17_, we performed Co K-edge X-ray absorption spectroscopy (XAS). The observed X-ray absorption near-edge structure (XANES) spectra are shown in Fig. 4b. The sharp lines correspond to the electron transition from Co 1*s* to outer unoccupied 4*p* orbitals. The energy position of the pre-edge peak and white line peak for both CoBDC and CoBDC-Fc_0.17_ are about at 7708.2 and 7723.6 eV, respectively. The spectral profile is very similar to that of CoO, indicating average Co valence state of +2^40^. In contrast, the peak intensity of CoBDC–Fc_0.17_ is slightly diminished relative to that of CoBDC, suggesting that introduction of the missing ligands changed the local coordination geometry of Co^2+^ ions^41^. The similar Co K-edge *k*^3^*χ* data of EXAFS oscillations are displayed from viewpoints of Cobalt (Fig. 4c), suggesting Co for CoBDC and CoBDC–Fc_0.17_ are in similar coordinated environments. Notably, CoBDC–Fc_0.17_ exhibits less amplitude oscillations, indicating the average coordination number of Co atom decreases after introducing missing linkers. To clarify the change of local coordination geometry of Co^2+^ ions observed in XANES, we performed the curve fitting of the Fourier transforms of the extended X-ray absorption fine structure (EXAFS) for CoBDC and CoBDC-Fc_0.17_ (Fig. 4d, Supplementary Table 4). The curve fitting revealed that the Co–O distance of CoBDC–Fc_0.17_ (2.08 Å) is almost identical to that of CoBDC (2.07 Å). On the other hand, the coordination number of Co–O for CoBDC–Fc_0.17_ was 4.4, which is smaller than that of CoBDC (6.2). These observations suggest introducing the missing linkers generated unsaturated Co^2+^ sites, which is expected to work as active sites for the OER.

### Electrocatalytic performance of missing-linker MOFs

**Fig. 5:**
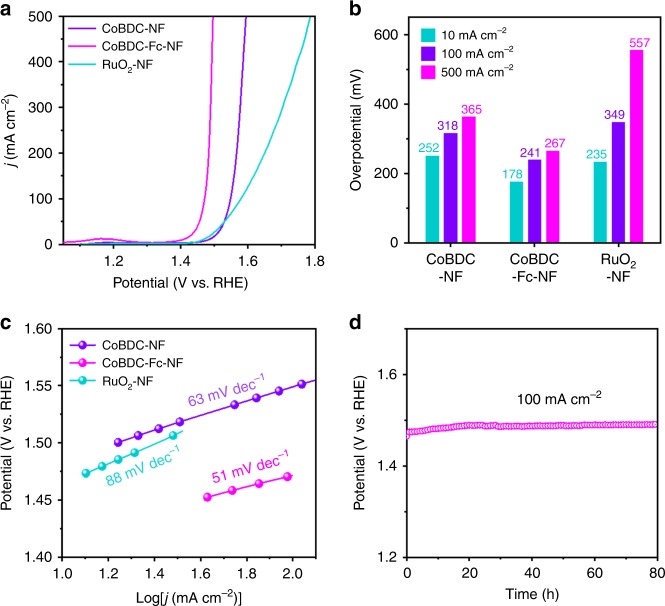
OER performance. **a** Linear sweep voltammetry curves toward OER. **b** Overpotential at different current densities. **c** Tafel plots of different catalysts. **d** Chronopotentiometry curves of CoBDC-Fc-NF for 80 h at 100 mA cm^−2^ in 1 M KOH

Next, we sought to verify the electronic structure change of Co active center by introducing missing linkers to facilitate OER performance. The electrocatalytic performance of as-prepared MOFs was investigated with three-electrode system in alkaline condition (1 M KOH) by using commercially RuO_2_ as reference. CoBDC and CoBDC–Fc_0.17_ were deposited onto a glassy-carbon electrode with a loading of 0.35 mg cm^−2^. CoBDC–NF and CoBDC–Fc–NF were directly used as a self-supported electrode. The loading mass of CoBDC and CoBDC–Fc onto CoBDC–NF and CoBDC–Fc–NF was about 2 mg cm^−2^. The polarization curves with iR-compensation were recorded by linear sweep voltammetry (LSV). As shown in Supplementary Fig. 10, the CoBDC existed low OER performance with overpotential of 378 mV at 10 mA cm^−2^. After introducing missing linkers, CoBDC–Fc_0.17_ showed improved catalytic activity with overpotential of 291 mV at 10 mA cm^−2^ and smaller Tafel slope (61 mV dec^−1^). The direct growth of MOF on NF will enhance transport kinetics and electrical contact. As can be seen in Fig. 5a, CoBDC–NF showed excellent electrocatalytic property for OER with an overpotential of 318 mV at the large current density of 100 mA cm^−2^, which is better than that of the commercial catalyst RuO_2_ (349 mV). After introducing miss linkers (Fc), CoBDC–Fc–NF showed significantly enhanced OER activity with the ultralow overpotential of 178 mV to achieve 10 mA cm^−2^ which is 74 and 57 mV lower than that of CoBDC–NF and commercial RuO_2_ (Fig. 5b), respectively. Furthermore, very small overpotentials of 241 and 267 mV can drive a high current density of 100 and 500 mA cm^−2^ for the CoBDC–Fc–NF electrode which competes to the best OER electrocatalysts previously reported (Supplementary Table 5)^42–46^. Additionally, CoBDC–Fc–NF has a highest turnover frequency (TOF) of 0.034 S^−1^ at an overpotential of 250 mV, further demonstrating its improved OER performance (Supplementary Fig. 11). The CoBDC–Fc–NF also shows smaller Tafel slope of 51 mV dec^−1^ compared with CoBDC-NF (63 mV dec^−1^) and RuO_2_ (88 mV dec^−1^), indicating more superior OER reaction kinetics. The stability of CoBDC-Fc-NF was tested by chronopotentiometry and cyclic voltammetry (CV). As shown in Fig. 5d, CoBDC–Fc–NF exhibited strong durability in a prolonged chronopotentiometry test at a constant current density of 100 mA cm^−2^ for 80 h. After CV between 1.20 and 1.45 V for 2000 cycles, CoBDC–Fc–NF only showed slight degradation, demonstrating its high durability (Supplementary Fig. 12). Additionally, the morphology and crystal structure of CoBDC–Fc–NF showed limited changes after 10 h electrocatalysis at a constant current density of 100 mA cm^−2^ (Supplementary Figs. 13 and 14). After 5 h electrocatalysis, the elemental composition of catalyst and chemical environment of Co had very little changes (Supplementary Fig. 15). XPS spectra of CoBDC–Fc–NF after 10 h electrocatalysis showed that the peak width and satellites intensity of Co 2*p* decreased slightly, indicating the formation of a small amount of Co^3+^ after long-time OER process (Supplementary Fig. 15)^47,48^. The peaks at binding energy of 779.8 and 795.0 eV are ascribed to Co^3+^ in CoOOH, confirming the formation of amorphous CoOOH during the long-time OER electrocatalytic process (Supplementary Figs. 13 and 15b)^47–49^. The limited changes in elemental composition and chemical environment of CoBDC–Fc–NF after 10 h OER stability test indicate the MOF to be the main component. Even so, the participation of CoOOH, widely known as an active species for OER electrocatalysis, cannot be excluded at this time.

The concentration of missing linkers can be controlled by varying the addition amount of Fc. A series of CoBDC–Fc_*x*_ powders with different ratios of BDC:Fc were prepared. SEM images showed that after introducing Fc into CoBDC the original morphology of nanosheet basically remains (Supplementary Fig. 16). The high crystallinity of CoBDC–Fc_*x*_ was evident from the XRD pattern, which was isostructural with CoBDC (Supplementary Fig. 17). Introducing Fc into CoBDC can obviously improve the electrocatalytic activity of MOFs (Supplementary Fig. 18). A doping ratio of 0.14 (Fc:BDC), exhibited optimal performance with an overpotential of 291 mV at 10 mA cm^−2^.

To demonstrate the universality of the missing linker engineering of MOF for OER, other monocarboxylic acid ligands including 4-nitrobenzoic acid (PNBA) and 4-carboxybenzaldehyde (PCBA) were used for preparing defective MOFs (CoBDC–PNBA, CoBDC–PCBA), which have the similar morphology with CoBDC (Supplementary Fig. 19). XRD pattern showed that CoBDC–PNBA and CoBDC–PCBA were isostructural with CoBDC (Supplementary Fig. 20). XPS showed that the Co 2*p*3/2 of CoBDC–PNBA and CoBDC–PCBA had higher binding energy and broadened peak than that of CoBDC (Supplementary Fig. 21a). Compared with that of CoBDC, the valence band maximum energy of CoBDC–PNBA and CoBDC–PCBA blue shifts to the vacuum level at about 1.60 and 1.48 eV, respectively, indicating the change of electronic structure of MOFs (Supplementary Fig. 21b). Encouraged by these results, we tested the OER performance of CoBDC–PNBA and CoBDC–PCBA. As shown in Supplementary Fig. 22, introduction of missing linkers significantly improved the catalytic properties. Moreover, CoBDC–PNBA and CoBDC–PCBA can grow in situ on NF to form uniform films (CoBDC–PNBA–NF, CoBDC–PCBA–NF) (Supplementary Fig. 23). The OER performance of CoBDC–PNBA–NF, CoBDC–PCBA–NF was apparently better than CoBDC–NF with overpotentials of 212 and 209 mV at 10 mA cm^−2^ (Supplementary Figs. 24a and 25). The low Tafel slopes of CoBDC-PNBA-NF (56 mV dec^−1^) and CoBDC–PCBA–NF (62 mV dec^−1^) indicated favorable kinetic process (Supplementary Fig. 24b). These results further demonstrate that tuning the electronic structure of MOFs by introducing missing linkers can enhance the electrocatalytic activity.

To gain more insights into the outstanding catalytic activity of defective MOFs, electrochemical impedance spectroscopy (EIS) techniques were used to investigate the kinetics of electrode reactions (Supplementary Fig. 26a). Introducing missing linkers into MOFs led to smaller charge-transfer resistance (*R*_ct_) for CoBDC-Fc-NF (2.21 Ω), CoBDC–PNBA–NF (4.95 Ω) and CoBDC–PCBA–NF (5.67 Ω) electrodes than that of CoBDC–NF electrodes (6.92 Ω), suggesting the lower activation energy for the reactions on MOFs with missing linkers (Supplementary Table 6). The electrochemically active surface area (ECSA) of as-prepared materials were also evaluated by the electrochemical double-layer capacitance (*C*_dl_) (Supplementary Fig. 27). As seen in Supplementary Fig. 26b, the *C*_dl_ of the sample of CoBDC–Fc–NF, CoBDC–PNBA–NF, and CoBDC–PCBA–NF is even lower than that of CoBDC–NF, indicating less electroactive surface of defective MOFs films. However, better electrocatalytic performance of missing-linker MOFs confirms that activity enhancement should be attributed to the increase of intrinsic activity of active sites by introducing missing linkers, which is consistent with DFT results.

## Discussion

In summary, we have developed a strategy to design efficient MOF electrocatalysts containing missing linkers. DFT predicted the regulation of electronic structure and OER activity in the MOF after introducing missing linkers. Subsequently, a series of MOFs with missing linkers can be successfully synthesized by modulation approach. The concentration of missing linkers can be well controlled by varying the addition amount of monocarboxylic acid. XPS and XAFS results verify the regulation of electronic structure after incorporating missing linkers into MOFs. The improved OER performance of missing-linkers MOFs is providing evidence that OER activity of MOFs can be indeed facilitated by modulating electronic structure via introducing missing linkers. Importantly, this study gives a new strategy to regulate the electronic structure of MOFs as high-efficiency electrocatalysts for potential applications.

## Methods

### Chemicals

Cobalt (II) nitrate hexahydrate (98%), terephthalic acid (99%), ferrocenecarboxylic acid (98%), PNBA (99%), 4-carboxylbenzaldehyde (99%), NaOH (97%), and commercial RuO_2_ (99.9%) were purchased from Aladdin (Shanghai, China). Solvents were purchased from commercial sources. NF was ultrasonically washed with HCl solution (3 M) for 30 min.

### Preparation of CoBDC–NF

Terephthalic acid (83 mg, 0.5 mmol) were dissolved in 5 mL N,N- dimethylformamide (DMF). Then, 1 mL 0.4 M NaOH was added under stirring. The solution above was slowly mixed with 5 mL cobalt (II) nitrate hexahydrate (145 mg, 0.5 mmol) DMF solution in a 30 mL Teflon-lined stainless-steel autoclave with a piece of NF (1 cm × 3 cm) in it. After that, the Teflon-lined stainless-steel autoclave was heated for 15 h at 100 °C. The resulting MOF film on NF was washed with DMF and ethanol three times and dried naturally. The loading amount of the MOF on NF was determined to be about 2.1 mg cm^−2^.

### Preparation of CoBDC–Fc–NF

Terephthalic acid (125 mg, 0.75 mmol) and ferrocenecarboxylic acid (23 mg, 0.1 mmol) were dissolved in 5 mL DMF. Then, 1 mL 0.4 M NaOH was added under stirring. After that, the solution above was slowly mixed with 5 mL cobalt (II) nitrate hexahydrate (218 mg, 0.75 mmol) DMF solution in a 30 mL Teflon-lined stainless-steel autoclave with a piece of NF (1 cm × 3 cm) in it. The Teflon-lined stainless-steel autoclave was heated for 15 h at 100 °C. The resulting MOF film on NF was washed with DMF and ethanol three times and dried naturally. The loading amount of the MOF on NF was determined to be about 1.8 mg cm^−2^.

### Preparation of CoBDC

Terephthalic acid (166 mg, 1 mmol) and cobalt (II) nitrate hexahydrate (291 mg, 1 mmol) were dissolved in 5 mL N,N-DMF, respectively. Then, the solution above was slowly mixed in a 30 mL Teflon-lined stainless-steel autoclave. After that, the Teflon-lined stainless-steel autoclave was heated for 12 h at 100 °C. The products were washed with DMF and methanol and dried at 60 °C for 12 h.

### Preparation of CoBDC–Fc_*x*_

Terephthalic acid (166 mg, 1 mmol) and various amounts of ferrocenecarboxylic acid were dissolved in 5 mL DMF. Then, the solution above was slowly mixed with 5 mL cobalt (II) nitrate hexahydrate (290 mg, 1 mmol) DMF solution in a 30 mL Teflon-lined stainless-steel autoclave. After that, the Teflon-lined stainless-steel autoclave was heated for 12 h at 100 °C. The resulting products were washed with DMF and ethanol three times and dried naturally. The content of Co and Fe was measured by inductively coupled plasma mass spectrometry (ICP–MS) to determine the ratio of terephthalic acid and ferrocenecarboxylic acid.

### Preparation of RuO_2_–NF

5 mg RuO_2_, 950 μL ethanol and 50 μL Nafion were mixed and dispersed by ultrasonic for 30 min. 200 μL RuO_2_ ink was depositing onto NF (1 cm × 0.5 cm) with a loading amount of 2 mg cm^−2^.

### Preparation of CoBDC–PNBA

Terephthalic acid (166 mg, 1 mmol) and PNBA (17 mg, 0.1 mmol) were dissolved in 5 mL DMF. Then, the solution above was slowly mixed with 5 mL cobalt (II) nitrate hexahydrate (290 mg, 1 mmol) DMF solution in a 30 mL Teflon-lined stainless-steel autoclave. After that, the Teflon-lined stainless-steel autoclave was heated for 12 h at 100 °C. The resulting products were washed with DMF and ethanol three times and dried naturally.

### Preparation of CoBDC–PCBA

Terephthalic acid (166 mg, 1 mmol) and 4-carboxylbenzaldehyde (15 mg, 0.1 mmol) were dissolved in 5 mL DMF. Then, the solution above was slowly mixed with 5 mL cobalt (II) nitrate hexahydrate (290 mg, 1 mmol) DMF solution in a 30 mL Teflon-lined stainless-steel autoclave. After that, the Teflon-lined stainless-steel autoclave was heated for 12 h at 100 °C. The resulting products were washed with DMF and ethanol three times and dried naturally.

### Preparation of CoBDC–PNBA–NF

Terephthalic acid (83 mg, 0.5 mmol) and PNBA (17 mg, 0.1 mmol) were dissolved in 5 mL DMF. Then, the solution above was slowly mixed with 5 mL cobalt (II) nitrate hexahydrate (145 mg, 0.5 mmol) DMF solution in a 30 mL Teflon-lined stainless-steel autoclave with a piece of NF (1 cm × 3 cm) in it. After that, the Teflon-lined stainless-steel autoclave was heated for 12 h at 100 °C. The resulting MOF film on NF was washed with DMF and ethanol three times and dried naturally. The loading amount of the MOF on NF was determined to be about 1.9 mg cm^−2^.

### Preparation of CoBDC–PCBA–NF

Terephthalic acid (83 mg, 0.5 mmol) and 4-carboxylbenzaldehyde (15 mg, 0.1 mmol) were dissolved in 5 mL DMF. Then, the solution above was slowly mixed with 5 mL cobalt (II) nitrate hexahydrate (145 mg, 0.5 mmol) DMF solution in a 30 mL Teflon-lined stainless-steel autoclave with a piece of NF (1 cm × 3 cm) in it. After that, the Teflon-lined stainless-steel autoclave was heated for 12 h at 100 °C. The resulting MOF film on NF was washed with DMF and ethanol three times and dried naturally. The loading amount of the MOF on NF was determined to be about 1.9 mg cm^−2^.

### Characterization

Powder XRD was measured on Rigaku SmartLab diffractometer with Cu Kα X-ray source (*λ* = 1.540598 Å). SEM images were measured with a Hitachi SU8010 system. TEM images were taken on a JEM-1400Plus TEM. STEM and EDX mapping images were recorded from a JEOL JEM-ARM200F equipped with energy-dispersive X-ray spectrometer, operating at 200 kV. XPS were performed on a VG Scientific ESCALAB 250 instrument. X-ray absorption spectra were collected at the BL14B2 beamline, SPring-8. in transmission mode under ambient conditions, using a Si (311) double crystal monochromator for Co *K*-edge. The data were processed with IFEFFIT^50^. Fourier transformation was *k*^3^-weighted in the *k* range from 2.9 to 11.7 Å^–1^. Gas sorption isotherms measurements were measured with a Micromeritics 3Flex Version 4.02 instrument. ICP–MS data were obtained from Thermo Scientific iCAP RQ instrument. Square resistance was measured with a FT-331 Series four-probe resistance ratio test instrument.

### Electrochemical measurements

Electrochemical measurements were performed in a three-electrode system controlled by a CHI 760E electrochemistry workstation. The Ag/AgCl and platinum plate electrode were used as the reference and counter electrode respectively. The measured potentials were converted vs. RHE, *E*_RHE_ = *E*_Ag/AgCl_ + 0.197 + 0.059 × pH. The MOF nanoarrays were directly used as a working electrode. LSV curves were recorded at a scan rate of 2 mV/s. The potential in the LSV polarization curves were corrected for iR losses. The ECSA were investigated by double-layer capacitance (*C*_dl_) in the potential range from 0.82 to 0.94 V vs. RHE. EIS were measured in a frequency range from 10^5^ to 0.01 Hz at 1.45 V vs. RHE with 5 mV amplitude. The TOF was calculated by the equation: TOF = (*J* × *A*)/(4 × *F* × *m*), where *J* represents the current density (A cm^−2^) at an overpotential of 250 mV, *A* and *m* represent the area of the electrode and the number of moles of the active materials.

### Computation method

All spin-polarized density-functional theory (DFT) calculations were performed using the Vienna ab initio simulation package (VASP)^51^. Electron–ion interactions were described using standard PAW potentials^52^. For the electron–electron exchange and correlation functional was described through the generalized gradient approximation of Perdew–Burke–Ernzerhof (PBE)^53^. A plane wave cutoff energy of 520 eV was applied in our calculations. Due to insufficient consideration of the on-site Columbic repulsion, between Co *d* electrons, the GGA + U approach was used with *U*−*J* = 4.0 for the Co atoms^54,55^.

To study the mechanism of OERs, a (3 × 2 × 2) supercell containing 264 atoms is used. The Brillouin-zone integrations were performed using the Gamma-point-only grid during the optimization. The iterative process considered was convergences, when the force on the atom was <0.02 eV Å^−1^ and the energy change was <10^–5^ eV per atom. The Gibbs free energy of each elementary step was calculated as

1 $$\Delta G = \Delta E + \Delta {\mathrm{{ZPE}}} - T \ {\bullet} \ \Delta S$$

where Δ*E* is the reaction energy calculated by the DFT method. ΔZPE and Δ*S* are the changes in zero-point energies and entropy during the reaction, respectively^56^.

## Supplementary information

Supplementary Information

Source data file

## Data Availability

Full data supporting the findings of this study are available within the article and its Supplementary Information, as well as from the corresponding author upon reasonable request.
