# Supplementary material for: Missing-linker metal-organic frameworks for oxygen evolution reaction
Source: Nat Commun. 2019 Nov 6;10:5048. doi: 10.1038/s41467-019-13051-2 (PMC6834668; doi:10.1038/s41467-019-13051-2)
Supplement: Supplementary file 1 — Supplementary Information [file 41467_2019_13051_MOESM1_ESM.pdf]

Supplementary Information

Missing-Linker Metal-organic Frameworks for Oxygen Evolution Reaction

Xue et al.

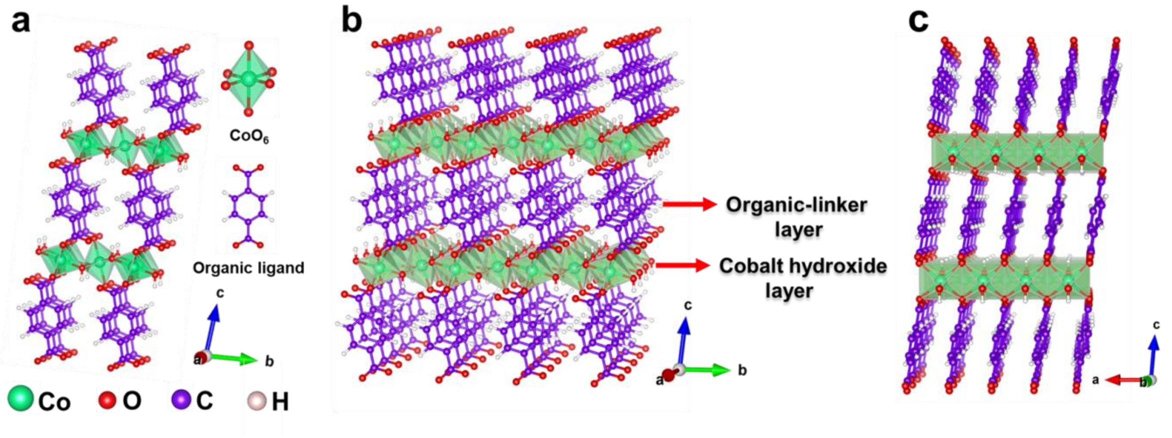

Supplementary Figure 1. Crystal structure of $Co_2(OH)_2(C_8H_4O_4)$ (named by CoBDC).

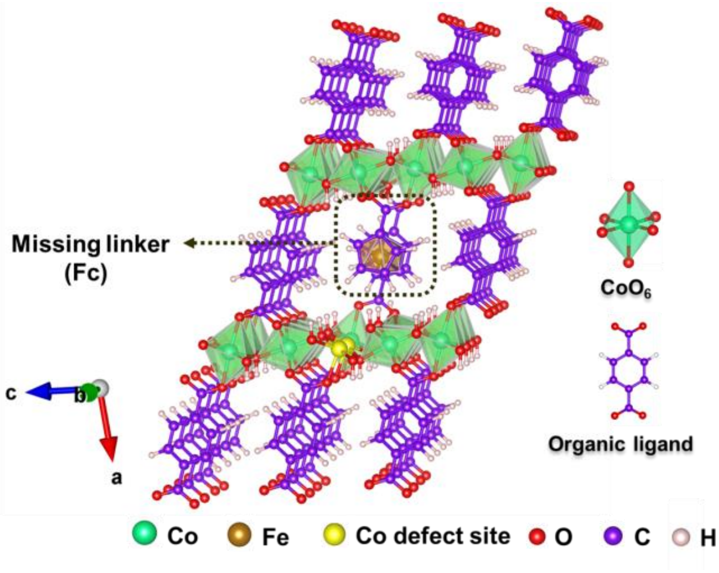

Supplementary Figure 2. Crystal structure of CoBDC-Fc derived from the known crystal structure of $Co_2(OH)_2(C_8H_4O_4)$.

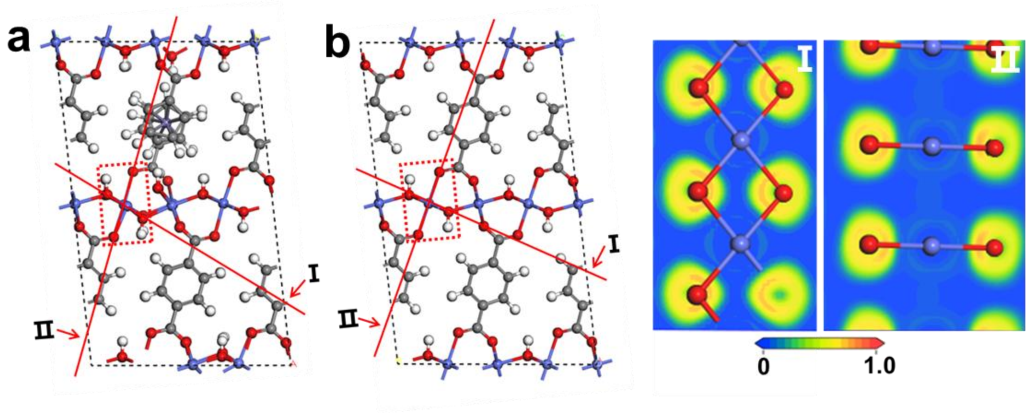

Supplementary Figure 3. Electron localization function. (a) Different sliced plane of CoBDC-Fc for electron localization calculation. (b) Electron localization function of CoBDC the different sliced plane.

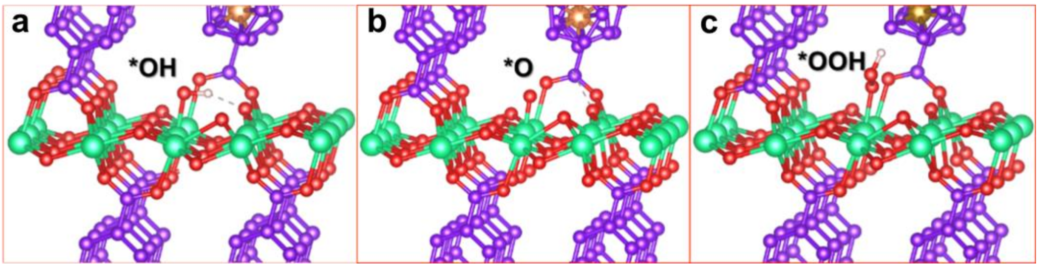

Supplementary Figure 4. Different adsorption intermediates for Co2 in CoBDC-Fc. (a) OH*; (b) O*; (c) OOH*.

Supplementary Table 1. The adsorption free energy at different sites for OER intermediates.

Active site	H_2O	OH*	O*	OOH*	O_2
**Co2 in CoBDC-Fc**	0	1.76	3.61	5.03	4.92
**Co1 in CoBDC-Fc**	0	3.74	5.51	6.90	4.92
**Co in CoBDC**	0	3.60	5.97	7.03	4.92

Supplementary Table 2. The molar ratio of BDC:Fc in different MOFs.

	**CoBDC-Fc$_{0.17}$**	**CoBDC-Fc$_{0.14}$**	**CoBDC-Fc$_{0.11}$**	**CoBDC-Fc$_{0.08}$**	**CoBDC-Fc-NF**
**Molar ratio (Co:Fe)[a]**	14:1	16:1	20	26:1	14:1
**Molar ratio (BDC:Fc)[b]**	6:1	7:1	9:1	12:1	6:1

[a] The molar ratio of Co:Fe was measured by inductively coupled plasma mass spectrometry (ICP-MS). [b] The molar ratio of BDC:Fc was determined by the molar ratio of Co:Fe and the structural formula of CoBDC ($Co_2(OH)_2(C_8H_4O_4)$).

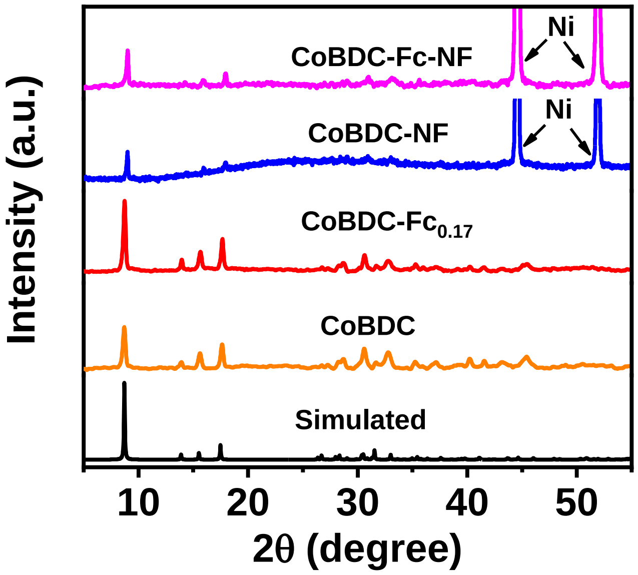

Supplementary Figure 5. XRD of CoBDC, CoBDC-Fc$_{0.17}$, CoBDC-NF and CoBDC-Fc-NF.

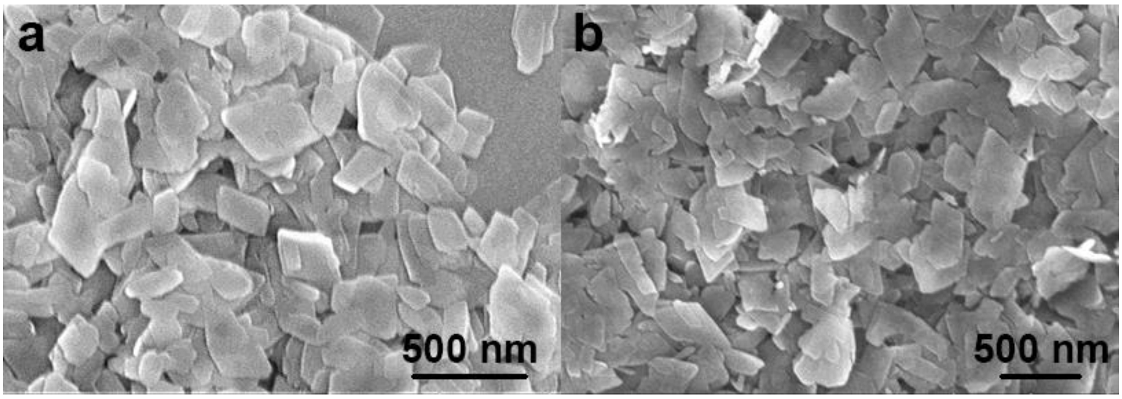

Supplementary Figure 6. SEM images of MOFs. (a) CoBDC; (b) CoBDC-$Fc_{0.17}$.

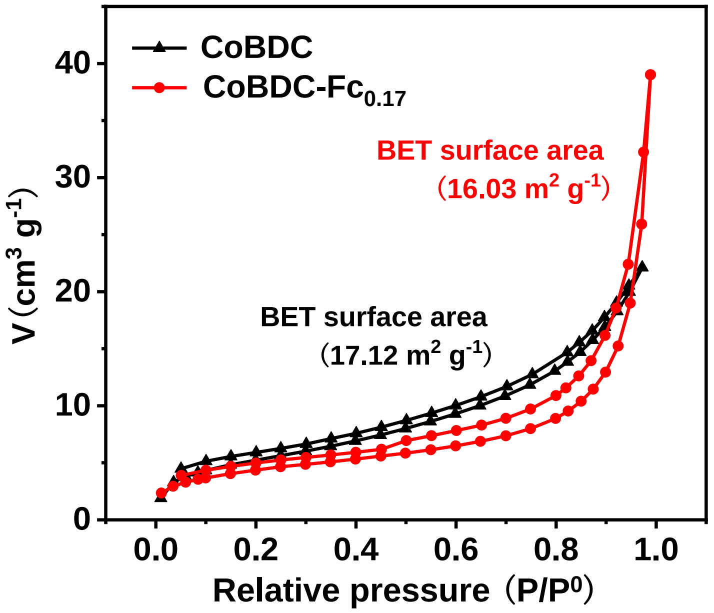

Supplementary Figure 7. N_2 adsorption-desorption isotherm of at 77K of CoBDC and CoBDC-$Fc_{0.17}$.

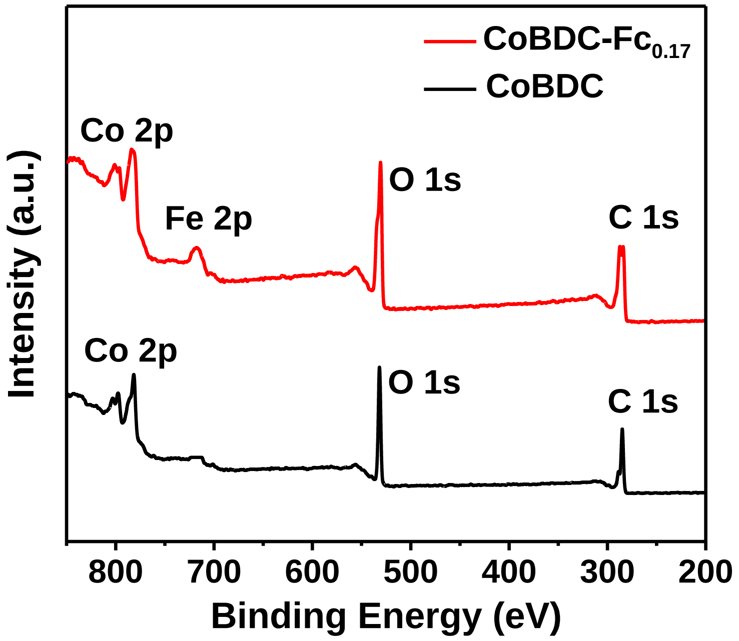

Supplementary Figure 8. Full range XPS spectra of CoBDC and CoBDC-$Fc_{0.17}$.

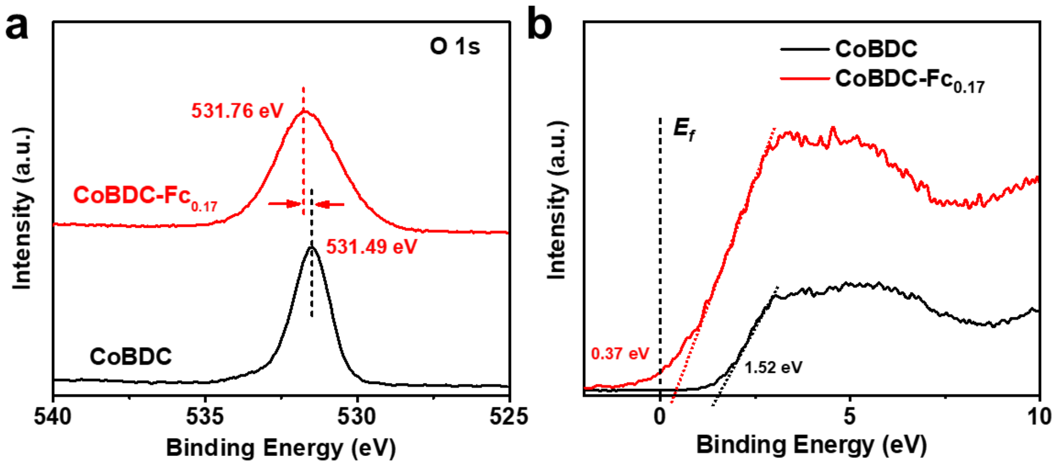

Supplementary Figure 9. XPS spectra of CoBDC and CoBDC-$Fc_{0.17}$. (a) O 1s spectra; (b) Valence band XPS data.

Supplementary Table 3. Square resistance of CoBDC, CoBDC-$Fc_{0.17}$, CoBDC-PCBA and CoBDC-PNBA.

**MOFs**	**CoBDC**	**CoBDC-$Fc_{0.17}$**	**CoBDC-PCBA**	**CoBDC-PNBA**
**Square resistance (kΩ/☐)**	57.2,	17.6	23.7	30.4

Supplementary Table 4. Fitting parameters of Co K-edge EXAFS curves for CoBDC and CoBDC-$Fc_{0.17}$.

Sample	Path	R (Å)[a]	N[b]	σ^2 (10^{-3}Å^2)[c]
CoBDC	Co-O	2.07±0.02	6.2±0.4	8.1±0.1
	Co-C/O	3.18±0.02	6.9±0.1	3.6±0.1
	Co-Co	3.56±0.02	5.6±0.7	4.9±0.1
CoBDC-$Fc_{0.17}$	Co-O	2.08±0.02	4.4±0.1	6.4±0.1
	Co-C/O	3.21±0.02	6.5±0.2	8.1±0.1
	Co-Co	3.56±0.01	5.6±0.5	6.4±0.1

[a] R: distance between absorber and backscatter atoms; [b] N: coordination number; [c] σ^2: Debye-Waller factor.

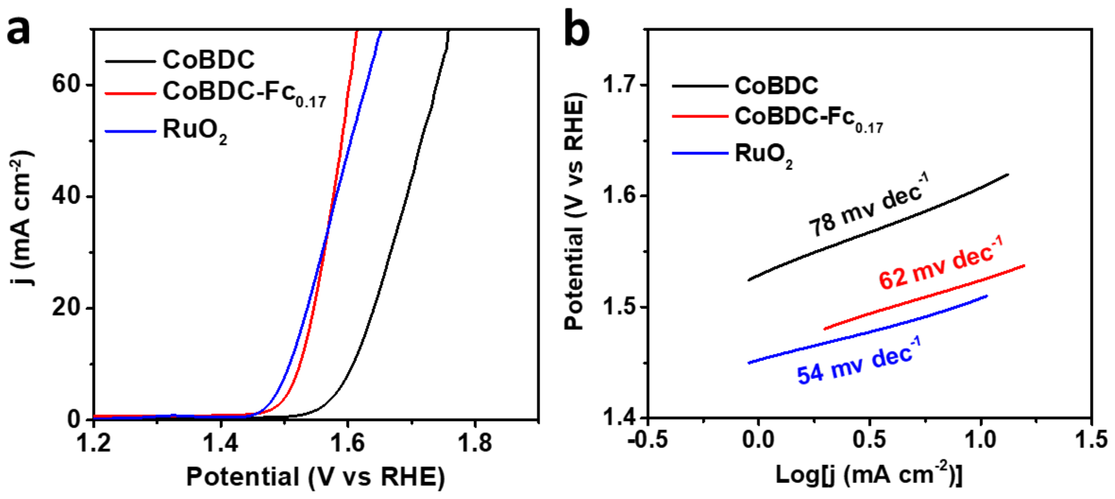

Supplementary Figure 10. OER performance of different catalysts. (a) Linear sweep voltammetry curves toward OER and (b) Tafel plots of different catalysts.

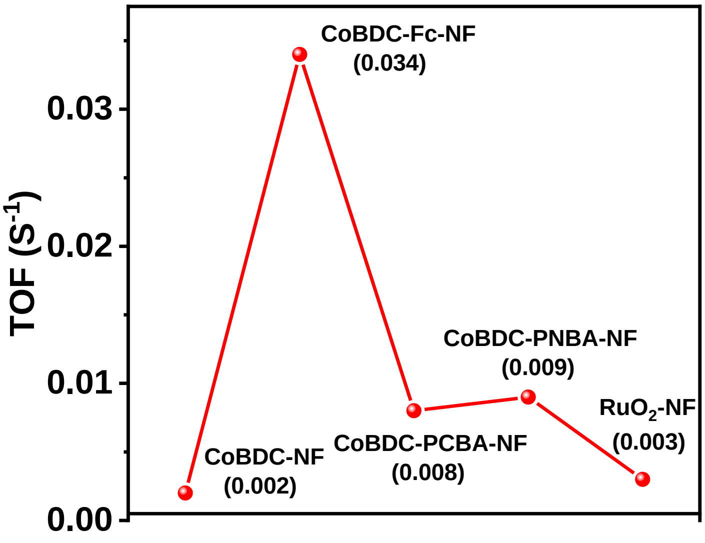

Supplementary Figure 11. Turnover frequency (TOF) of different materials at an overpotential of 250 mV.

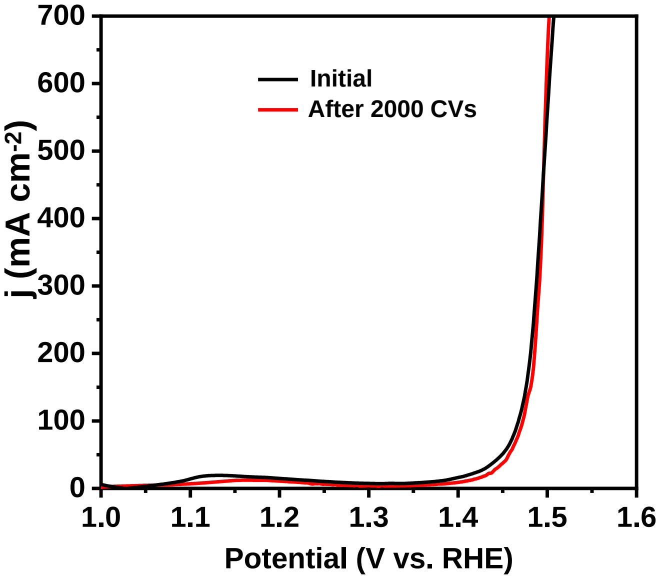

Supplementary Figure 12. Linear sweep voltammetry curves toward OER of CoBDC-Fc-NF after cyclic voltammetry between 1.20 V and 1.45 V for 2000 cycles.

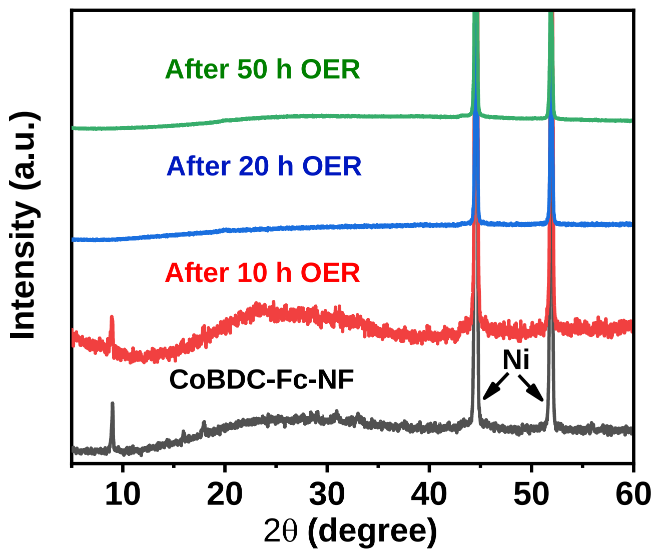

Supplementary Figure 13. XRD patterns of CoBDC-Fc-NF before and after OER process.

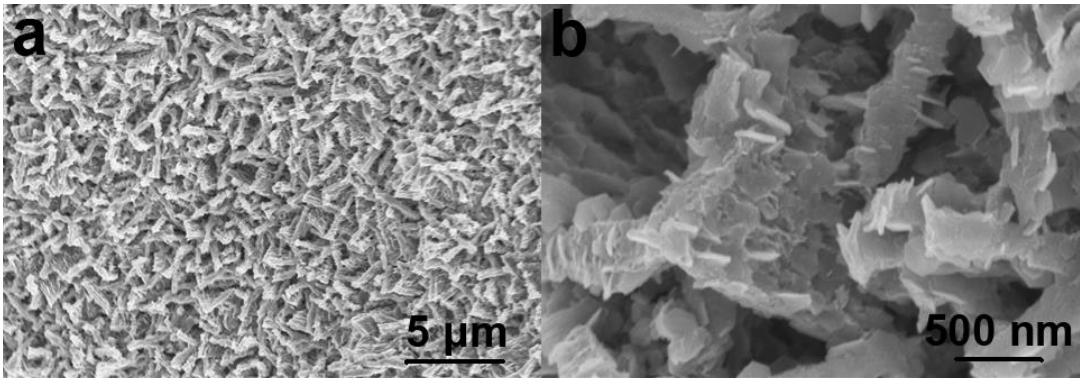

Supplementary Figure 14. SEM images of CoBDC-Fc-NF after 10 h electrocatalysis at a constant current density of 100 mA cm^{-2}. The morphology of nanoarray was mostly remained but with coarser surface.

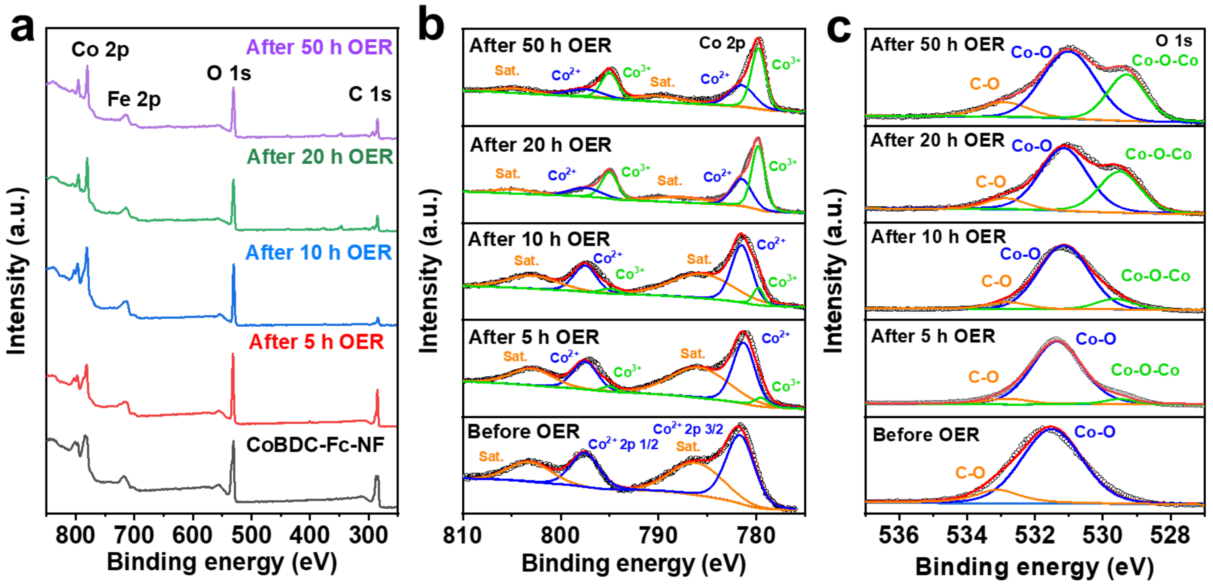

Supplementary Figure 15. XPS spectra of CoBDC-Fc-NF after electrocatalysis at a constant current density of 100 mA cm^{-2}.

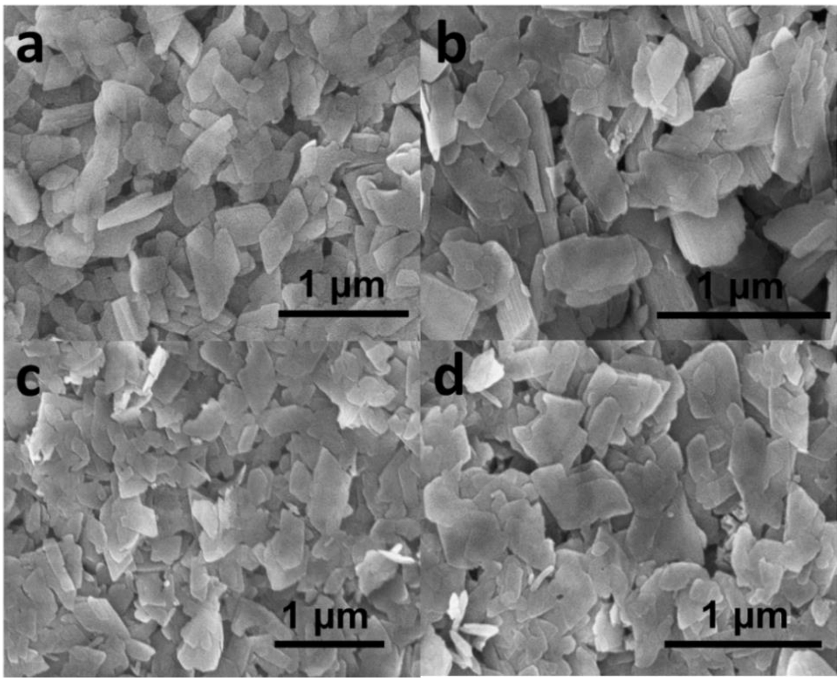

Supplementary Figure 16. SEM images of different MOFs. (a) CoBDC-$Fc_{0.08}$; (b) CoBDC-$Fc_{0.11}$; (c) CoBDC-$Fc_{0.14}$ and (d) CoBDC-$Fc_{0.17}$.

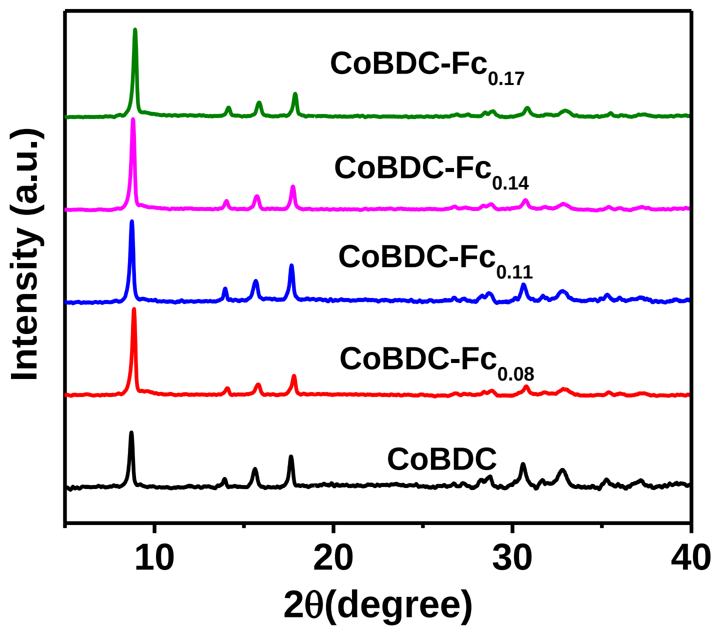

Supplementary Figure 17. PXRD patterns of different MOFs.

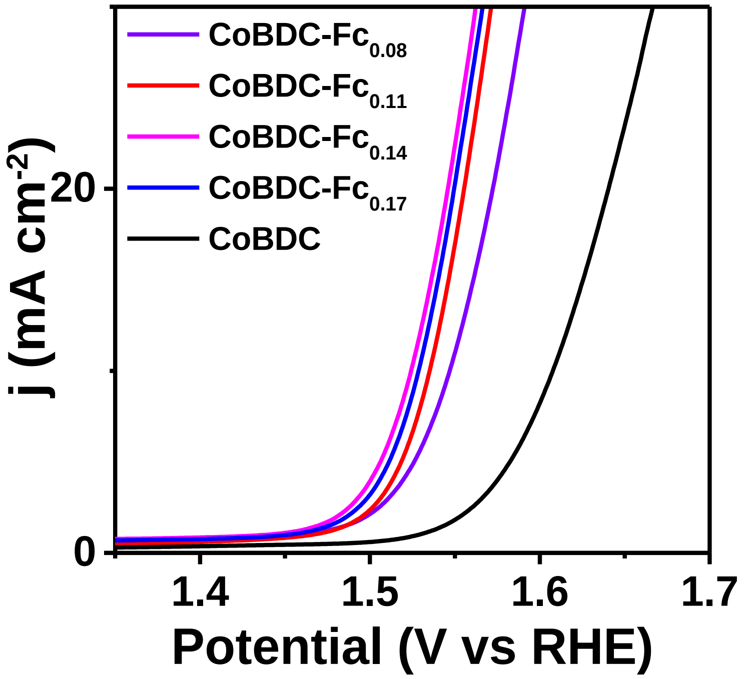

Supplementary Figure 18. Linear sweep voltammetry curves toward OER of different catalysts.

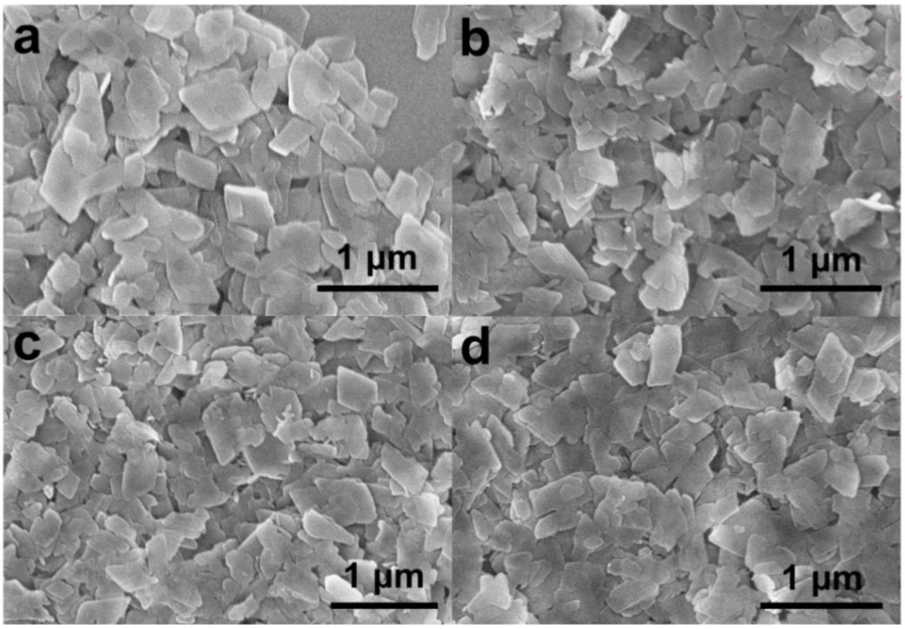

Supplementary Figure 19. SEM images of different MOFs. (a)CoBDC; (b) CoBDC-Fc; (c) CoBDC-PNBA; (d) CoBDC-PCBA.

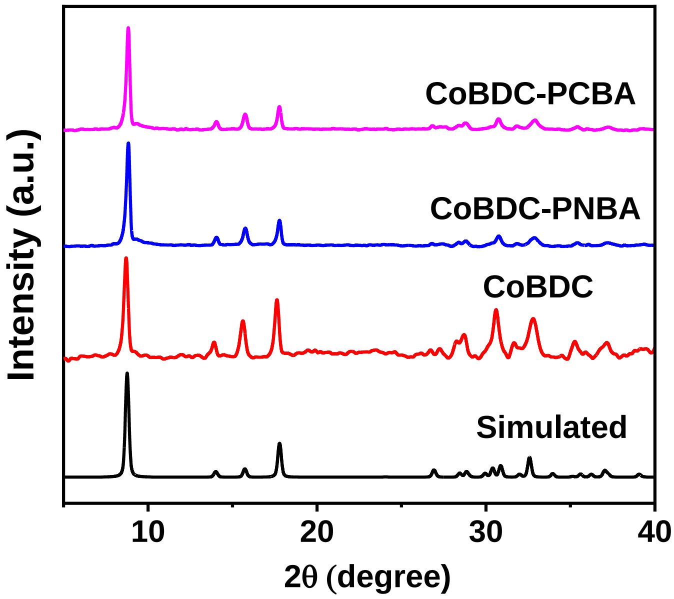

Supplementary Figure 20. PXRD patterns of different MOFs.

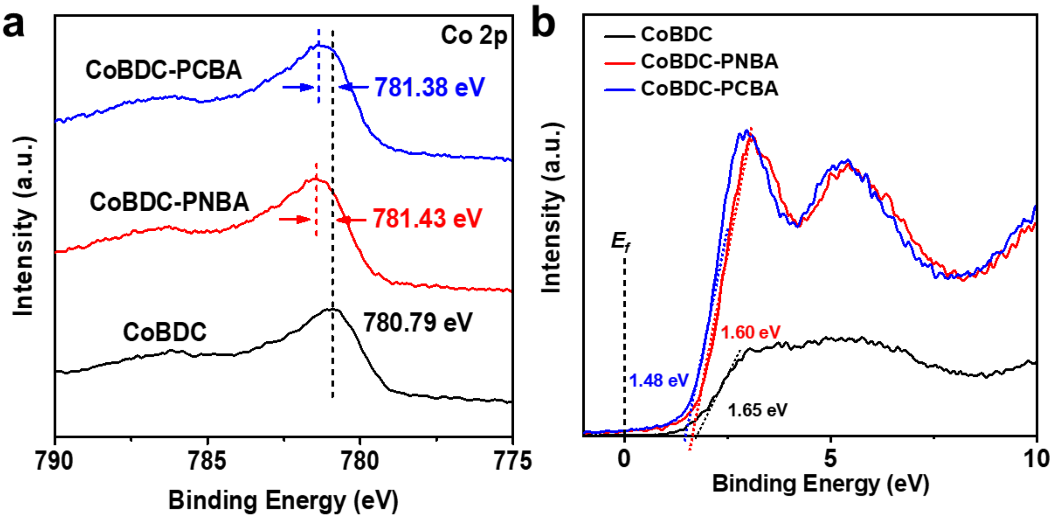

Supplementary Figure 21. XPS spectra of CoBDC, CoBDC-PNBA and CoBDC-PCBA. (a) Co 2p 3/2 spectra; (b) Valence band XPS data.

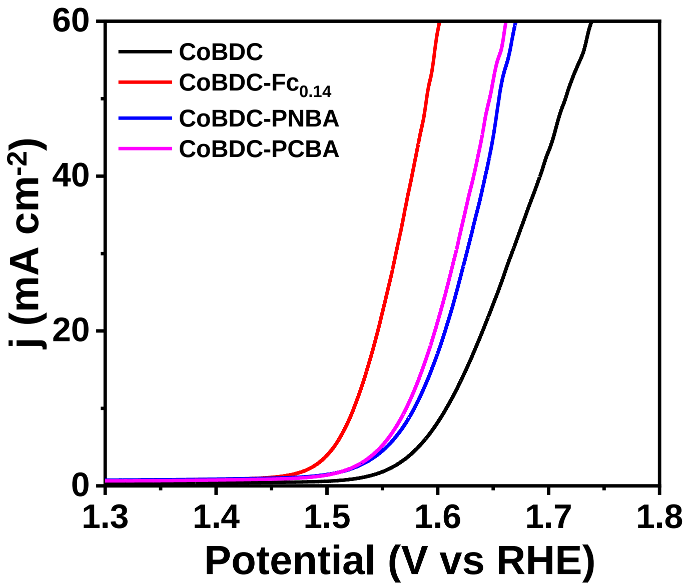

Supplementary Figure 22. Linear sweep voltammetry curves toward OER of CoBDC, CoBDC-$Fc_{0.14}$, CoBDC-PNBA and CoBDC-PCBA.

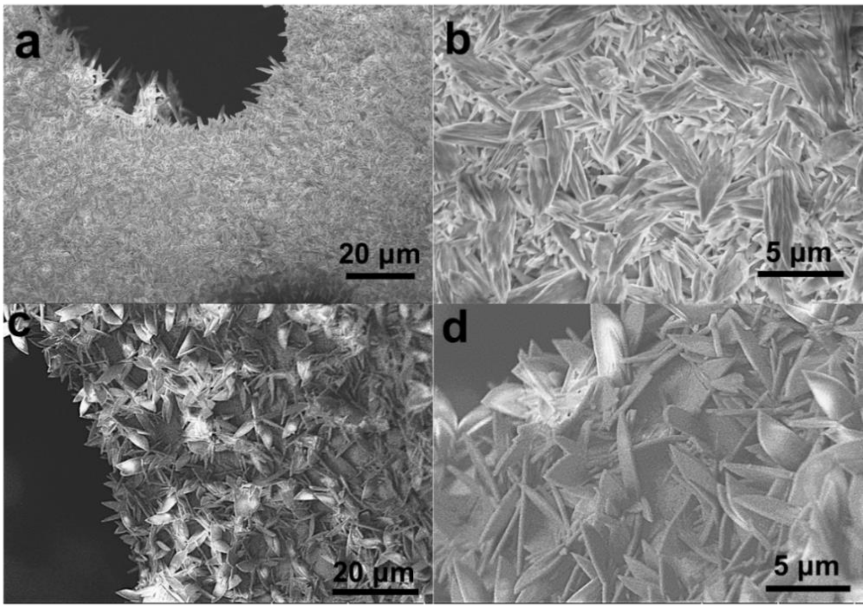

Supplementary Figure 23. SEM images of (a) and (b) CoBDC-PNBA-NF, (c) and (d) CoBDC-PCBA-NF.

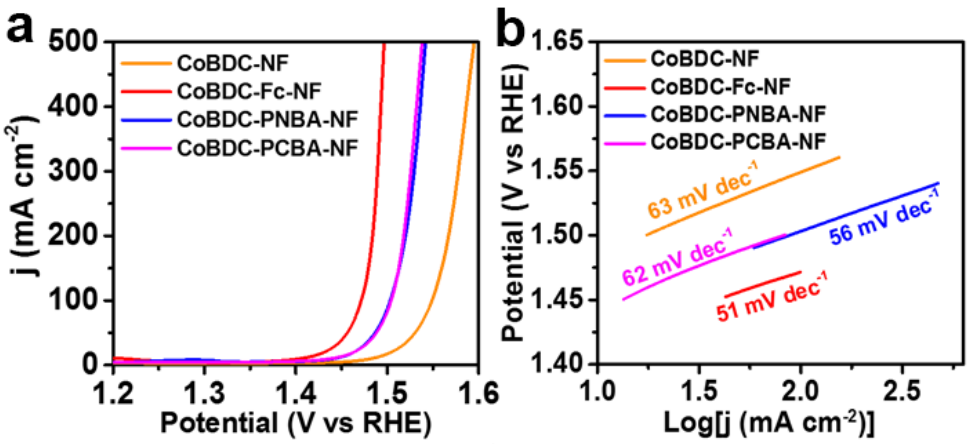

Supplementary Figure 24. OER performance of different missing-linker MOFs. (a) Linear sweep voltammetry curves of different catalysts, (b) Tafel plots of different catalysts.

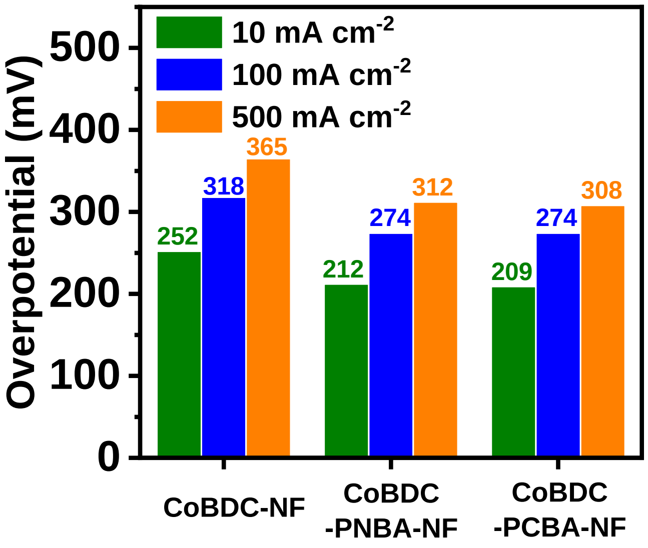

Supplementary Figure 25. The corresponding overpotentials of different catalysts at 10 mA cm^{-2}, 100 mA cm^{-2} and 500 mA cm^{-2}.

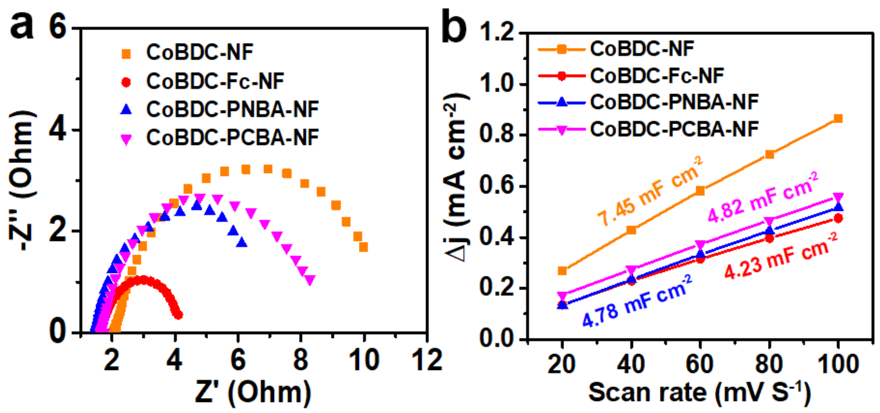

Supplementary Figure 26. Electrochemical impedance spectra and double layer capacitance of different electrode materials. (a) Electrochemical impedance spectra; (b) Double-layer capacitance.

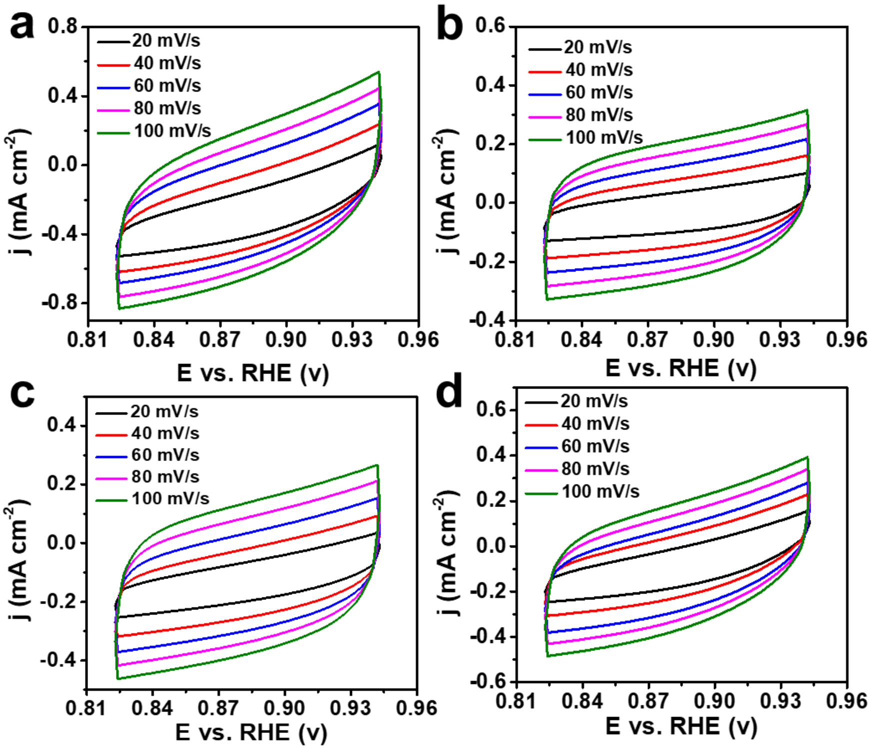

Supplementary Figure 27. CV plots of different electrode materials. (a) CoBDC-NF; (b) CoBDC-Fc-NF; (c) CoBDC-PNBA-NF; (d) CoBDC-PCBA-NF at different scan rate. The current at 0.88 V were as a function of scan rate to obtain the C_{dl}.

Supplementary Table 5. Comparisons of OER activity of Co-based electrocatalysts.

catalyst	Electrolyte	Overpotential at 10 mA cm^{-2} (mV)	Tafel slope (mV dec^{-1})	Substrates	Reference
CoBDC-Fc-NF	1.0 M KOH	178 at 10 mA cm^{-2}<br>241 at 100 mA cm^{-2}	51	Ni foam	This work
MAF-X27-OH	1.0 M KOH	387	60	GCE	1
Co-WOC-1	0.1 M KOH	390 at 1 mA cm^{-2}	128	GCE	2
FeTPyP-Co	0.1 M NaOH	351 at 1 mA cm^{-2}	NA	Au	3
Co-Nx/C NRA	0.1 M KOH	300	62.3	Ti-foil	4
$Co(OH)_2$	1.0 M KOH	470	NA	Ni foam	5
Co_3O_4@C-MWCNTs.	1.0 M KOH	320	62	GCE	6
$Co(OH)_2$	1.0 M NaOH	360	56	Au	7
CoMn LDH	1.0 M KOH	324	43	GCE	8
LDH FeCo	1.0 M KOH	330	85	GCE	9
NiCo-UMOFNs	1.0 M KOH	250	42	GCE	10
CoCo LDH	1.0 M KOH	393	59	GCE	11
CoCd-MOF	0.1 M KOH	353 at 1 mA cm^{-2}	110	GCE	12
$Zn_{0.1}Co_{0.9}Se_2$	1.0 M KOH	340	43.2	GCE	13
NCF-MOF	1.0 M KOH	320	49	GCE	14
Co/Co_3O_4@PGS	1.0 M KOH	350	76.1	GCE	15
$Ni_{0.6}Co_{1.4}P$	1.0 M KOH	300	80	GCE	16
$Co_5Mo_{1.0}O$ NSs@NF	1.0 M KOH	270	54.4	Ni foam	17
$Fe_{0.33}Co_{0.67}OOH$ PNSAs/CFC	1.0 M KOH	266	30	carbon fiber cloth	18
Co_1Mn_1CH/NF	1.0 M KOH	349 at 100 mA cm^{-2}	NA	Ni foam	19
$W_{0.5}Co_{0.4}Fe_{0.1}$/NF	1.0 M KOH	310 at 100 mA cm^{-2}	32	Ni foam	20
Co_3S_4@MoS_2	1.0 M KOH	280	43	GCE	21
O-Co_3O_4	1.0 M KOH	220	49.1	Ni foam	22
Fe‐Co‐P	1.0 M KOH	252	33	GCE	23
CoFeZr oxides/NF	1.0 M KOH	248	54.2	Ni foam	24
$Zn_{0.35}Co_{0.65}O$	1.0 M KOH	322	42.6	GCE	25
NiCoFe@NiCoFeO NTAs/CFC	1.0 M KOH	201	39	carbon fiber cloth	26
Co@N‐CS/N‐HCP@CC	1.0 M KOH	248	68	carbon fiber cloth	27
Co_3S_4/EC‐MOF	1.0 M KOH	226	132	carbon fiber cloth	28
Co/β-Mo_2C@N-CNTs	1.0 M KOH	356	67	GCE	29

Supplementary Table 6. EIS results of CoBDC-NF, CoBDC-Fc-NF, CoBDC-PNBA-NF and CoBDC-PCBA-NF.

catalyst	Solution series resistances Rs (Ω)	Charge transfer resistance R_{ct} (Ω)
**CoBDC**	2.07	6.92
**CoBDC-Fc-NF**	1.96	2.21
**CoBDC-PNBA-NF**	1.94	4.95
**CoBDC-PCBA-NF**	1.89	5.67

Supplementary References

1 Lu, X. F. *et al.* An Alkaline-Stable, Metal Hydroxide Mimicking Metal-Organic Framework for Efficient Electrocatalytic Oxygen Evolution. *J. Am. Chem. Soc.* **138**, 8336-8339 (2016).

2 Manna, P., Debgupta, J., Bose, S. & Das, S. K. A Mononuclear CoII Coordination Complex Locked in a Confined Space and Acting as an Electrochemical Water-Oxidation Catalyst: A “Ship-in-a-Bottle” Approach. *Angew. Chem. Int. Ed.* **55**, 2425-2430 (2016).

3 Wurster, B., Grumelli, D., Hotger, D., Gutzler, R. & Kern, K. Driving the Oxygen Evolution Reaction by Nonlinear Cooperativity in Bimetallic Coordination Catalysts. *J. Am. Chem. Soc.* **138**, 3623-3626 (2016).

4 Amiinu, I. S. et al. From 3D ZIF Nanocrystals to Co–Nx/C Nanorod Array Electrocatalysts for ORR, OER, and Zn-Air Batteries. *Adv. Funct. Mater.* **28**, 1704638 (2018).

5 Li, Y., Zhang, L., Xiang, X., Yan, D. P. & Li, F. Engineering of ZnCo-layered double hydroxide nanowalls toward high-efficiency electrochemical water oxidation. *J. Mater. Chem. A* **2**, 13250-13258 (2014).

6 Li, X. Z. et al. MOF derived Co_3O_4 nanoparticles embedded in N-doped mesoporous carbon layer/MWCNT hybrids: extraordinary bi-functional electrocatalysts for OER and ORR. *J. Mater. Chem. A* **3**, 17392-17402 (2015).

7 Abu Sayeed, M., Herd, T. & O'Mullane, A. P. Direct electrochemical formation of nanostructured amorphous $Co(OH)_2$ on gold electrodes with enhanced activity for the oxygen evolution reaction. *J. Mater. Chem. A* **4**, 991-999 (2016).

8 Song, F. & Hu, X. L. Ultrathin Cobalt-Manganese Layered Double Hydroxide Is an Efficient Oxygen Evolution Catalyst. *J. Am. Chem. Soc*. **136**, 16481-16484 (2014).

9 Zhang, B. et al. Homogeneously dispersed multimetal oxygen-evolving catalysts. *Science* **352**, 333-337 (2016).

10 Zhao, S. L. et al. Ultrathin metal-organic framework nanosheets for electrocatalytic oxygen evolution. *Nat. Energy* **1**, 1-10 (2016).

11 Song, F. & Hu, X. L. Exfoliation of layered double hydroxides for enhanced oxygen evolution catalysis. *Nat. Commun.* **5**, 4477 (2014).

12 Maity, K., Bhunia, K., Pradhan, D. & Biradha, K. Co(II)-Doped Cd-MOF as an Efficient Water Oxidation Catalyst: Doubly Interpenetrated Boron Nitride Network with the Encapsulation of Free Ligand Containing Pyridine Moieties. *ACS Appl. Mater. Inter.* **9**, 37548-37553 (2017).

13 Wang, X. et al. Hollow bimetallic cobalt-based selenide polyhedrons derived from metal-organic framework: an efficient bifunctional electrocatalyst for overall water splitting. *J. Mater. Chem. A* **5**, 17982-17989 (2017).

14 Ahn, W. et al. Hollow Multivoid Nanocuboids Derived from Ternary Ni-Co-Fe Prussian Blue Analog for Dual-Electrocatalysis of Oxygen and Hydrogen Evolution Reactions. *Adv. Funct. Mater.* **28**, 1802129 (2018).

15 Jiang, Y. et al. Interpenetrating Triphase Cobalt-Based Nanocomposites as Efficient Bifunctional Oxygen Electrocatalysts for Long-Lasting Rechargeable Zn-Air Batteries. *Adv. Energy. Mater*. **8**, 1702900 (2018).

16 Qiu, B. C. et al. Fabrication of Nickel-Cobalt Bimetal Phosphide Nanocages for Enhanced Oxygen Evolution Catalysis. *Adv. Funct. Mater*. **28**, 1706008 (2018).

17 Zhang, Y., Shao, Q., Long, S. & Huang, X. Q. Cobalt-molybdenum nanosheet arrays as highly efficient and stable earth-abundant electrocatalysts for overall water splitting. *Nano Energy* **45**, 448-455 (2018).

18 Ye, S. H., Shi, Z. X., Feng, J. X., Tong, Y. X. & Li, G. R. Activating CoOOH Porous Nanosheet Arrays by Partial Iron Substitution for Efficient Oxygen Evolution Reaction. *Angew. Chem. Int. Edit.* **57**, 2672-2676 (2018).

19 Tang, T. et al. Electronic and Morphological Dual Modulation of Cobalt Carbonate Hydroxides by Mn Doping toward Highly Efficient and Stable Bifunctional Electrocatalysts for Overall Water Splitting. *J. Am. Chem. Soc.* **139**, 8320-8328 (2017).

20 Pi, Y. C. et al. Trimetallic Oxyhydroxide Coralloids for Efficient Oxygen Evolution Electrocatalysis. *Angew. Chem. Int. Edit.* **56**, 4502-4506 (2017).

21 Guo, Y. N. et al. Elaborately assembled core-shell structured metal sulfides as a bifunctional catalyst for highly efficient electrochemical overall water splitting. *Nano. Energy*. **47**, 494-502 (2018).

22 Cai, Z. et al. Single-Crystalline Ultrathin Co_3O_4 Nanosheets with Massive Vacancy Defects for Enhanced Electrocatalysis. *Adv. Energy. Mater.* **8**, 1701694 (2018).

23 Liu, K. W. et al. High-Performance Transition Metal Phosphide Alloy Catalyst for Oxygen Evolution Reaction. *ACS Nano*. **12**, 158-167 (2018).

24 Huang, L. L. et al. Zirconium-Regulation-Induced Bifunctionality in 3D Cobalt-Iron Oxide Nanosheets for Overall Water Splitting. *Adv. Mater.* **31**, 1901439 (2019).

25 Wahl, S. et al. Zn0.35Co0.65O - A Stable and Highly Active Oxygen Evolution Catalyst Formed by Zinc Leaching and Tetrahedral Coordinated Cobalt in Wurtzite Structure. *Adv. Energy. Mater.* **9**, 1900328 (2019).

26 Liu, Y. et al. Valence Engineering via Selective Atomic Substitution on Tetrahedral Sites in Spinel Oxide for Highly Enhanced Oxygen Evolution Catalysis. *J. Am. Chem. Soc.* **141**, 8136-8145 (2019).

27 Chen, Z. L. et al. Oriented Transformation of Co-LDH into 2D/3D ZIF-67 to Achieve Co-N-C Hybrids for Efficient Overall Water Splitting. *Adv. Energy. Mater.* **9**, 1803918 (2019).

28 Liu, T. et al. Self-Sacrificial Template-Directed Vapor-Phase Growth of MOF Assemblies and Surface Vulcanization for Efficient Water Splitting. *Adv. Mater.* **31**, 1806672 (2019).

29 Ouyang, T., Ye, Y. Q., Wu, C. Y., Xiao, K. & Liu, Z. Q. Heterostructures Composed of N-Doped Carbon Nanotubes Encapsulating Cobalt and beta-Mo2C Nanoparticles as Bifunctional Electrodes for Water Splitting. *Angew. Chem. Int. Edit.* **58**, 4923-4928 (2019).
